# Psychological Consequences of Experiencing Violence in Childhood—The Role of Dissociation in the Formation of Early Maladaptive Schemas

**DOI:** 10.3390/jcm11174996

**Published:** 2022-08-25

**Authors:** Michał M. Sieński, Michał Ziarko

**Affiliations:** Faculty of Psychology and Cognitive Science, Adam Mickiewicz University, 60-568 Poznan, Poland

**Keywords:** violence, trauma, dissociation, early maladaptive schemas

## Abstract

Childhood experiences of violence can lead to severe psychological consequences. One of them is an increased risk of abnormal personality development. It can manifest as rigid negativistic beliefs about the self, others, and the surrounding world, which some specialists term early maladaptive schemas. The conducted study aims to provide a possible explanation of the role dissociation plays in the process of forming early maladaptive schemas. The study was conducted on 342 adult women whose biographies contained one or more episodes of potentially traumatic experiences of violence in childhood. Three questionnaires were used: Childhood Trauma Questionnaire, Dissociative Experiences Scale, Young Schema Questionnaire. The results show that experiences of violence are linked with dissociative disorders and the intensity of early maladaptive schemas. Mediation analysis confirmed that the relationship between experiencing violence and early maladaptive schemas is exacerbated by the presence of dissociative symptoms resulting from the violence experienced. The mediation analysis results suggest that if an experience of violence is followed by the emergence of dissociative symptoms, its impact on personality is more severe. This confirms earlier notions regarding the role that dissociative symptoms play in personality changes stemming from traumatic events.

## 1. Introduction

Jeffrey Young’s theory of early maladaptive schemas provides a psychological conceptualization of personality and behavioural disorders as well as a framework to design appropriate psychotherapeutic interventions. This approach assumes that negative experiences from early stages of life are recorded in an individual’s mind as certain cognitive and emotional models, which the authors term early maladaptive schemas (EMS). Unfavourable circumstances of an individual’s growth and development during the early stages of their life carry the risk of disrupting the development of their personality; the less favourable the circumstances, including the occurrence of traumatic events and serious neglect, the higher the risk of abnormal personality development, i.e., higher risk of more prominent formation of early maladaptive schemas. An EMS remains relatively stable over time, in particular due to the ability to process any event in such a way as to prove its core beliefs, making them resistant to change [1] Authors of the early maladaptive schema concept have presented 18 distinct schemas, separated into five general domains describing different aspects of experiencing the self, others, and the surrounding world: disconnection and rejection, impaired autonomy and performance, impaired limits, other-directedness, and overvigilance and inhibition [2].

Research on patients suffering from personality disorders affirms the general theoretical assumptions regarding the connection between early traumatic events and the development of personality and behavioural disorders [3,4,5,6,7]. It has been observed that biographies of patients with severe personality disorders (such as borderline personality disorder) include victimization by violence or severe neglect in their family of origin more often than those in other groups [3,5,7]. The link between enduring violence and abnormal personality development has been confirmed by empirical research results [4,6]. 

Numerous studies suggest that the theory of early maladaptive schemas has its use in both diagnosing different disorders, especially personality disorders [8,9,10], and explaining the consequences of experiencing traumatic events [11,12]. However, the psychological mechanism responsible for EMS forming after experiences of violence in childhood remains unclear. An unanswered key question is what factors take part in this process. Specifically, which of those factors protect against emerging EMS and which exacerbate them.

It is suspected that mechanisms related to dissociative disorders may be significant to the negative outcomes of early traumatic events.

Symptoms of dissociative disorders subsequent to a traumatic experience, especially ones such as depersonalization and derealization, lead to difficulties in perceiving, appraising, emotionally processing, remembering, and integrating lived events into the individual’s own identity. These symptoms can severely hinder adequate assessments of reality, including judgements of the potential risk involved in everyday situations [13,14,15]. Taking this into consideration, it seems probable that if an experience of childhood violence results in dissociative disorders, these disorders can play a significant role in the process of consolidating rigid beliefs about the self and the world, which eventually become early maladaptive schemas. On the basis of the early maladaptive schema theory, it is suspected that such a case reinforces schemas from the disconnection and rejection domain in particular, which transfers to the following attitudes towards the self, others, and the surrounding world:

- perceiving others as incapable of showing support and emotional closeness; believing that close ones cannot be depended upon, as they are unpredictable and emotionally unstable; fearing abandonment or being deprived of care and support;
- expecting deliberate harm and exploitation;
- being convinced of one’s own defectiveness, which causes a feeling of inferiority, of being bad and unwanted; fearing losing love when the defect is found out; feeling shame due to seeing one’s traits as flaws;
- believing in one’s alienation, otherness, and lack of belonging [2].

Assuming that other life events are not equally negative, exhibiting dissociative symptoms in the aftermath of trauma makes it more difficult to remember, ascribe emotional meaning to, and integrate new experiences which could in some way alter the previously formed image of the self, others, and the world. An example would be adding nuance by considering that people differ from one another and there are those who will not harm or exploit. Dissociative disorders, especially those of a persistent nature, as in PTSD sufferers, will effectively prevent this process. As a result of dissociative disorders, experiences potentially able to change the content of existing cognitive schemas may be ignored or marginalized in the patient’s general pool of experiences [13,16].

These circumstances lead us to formulate two research questions: (1) Is there a relationship between experiencing violence in childhood and the development of dissociative disorder symptoms and early maladaptive schemas? (2) What is the role of dissociative disorders in the process of early maladaptive schema formation in individuals who had experienced violence in childhood?

## 2. Materials and Methods

This work was created as part of research project no. 2016/21/N/HS6/02824 financed by the National Science Centre. Measurements taken in 2019–2022 were used.

Participants: The study was conducted on adult women whose biographies contain one or more episodes of potentially traumatic violence in close relationships. 141 of study participants experienced violence in childhood before the onset of adolescence (i.e., before the age of 11), while 201 participants experienced violence in both childhood and adulthood. A comparative analysis of participants with different life experiences was conducted to verify the similarity of childhood experiences, which are key to studying the determinants of the formation of early maladaptive schemas (Table 1). The only difference was observed in severity of physical neglect (U = 7.19; *p* < 0.05; d = 0.23).

Using a group that is homogenous in terms of gender is significant in light of conclusions from previous research, which indicated that long-term effects of experiencing violence in childhood may be different for men and women [17]. A total of 342 women were studied. Their average age was 35.8 years and the majority had a high-school level education (29.8%; *N* = 102). Paid work (39.2%; *N* = 134) and current unemployment (25.1%; *N* = 86) were the predominant employment status (Table 2).

Measures: Three questionnaires were used during the study.

The Childhood Trauma Questionnaire (CTQ) [18]. The CTQ measures 5 types of violence against children (emotional abuse, physical abuse, sexual abuse, emotional neglect, physical neglect). Participants respond to each item using a 5-point scale marking frequency (1—never; 5—very often). Item example: “I had to wear dirty clothes.” The instrument has demonstrated satisfactory psychometrical parameters of reliability (α = 0.79–0.96) (Bernstein, Fink, 1998). The questionnaire used examined the presence of symptoms of dissociative disorders after violence experienced in childhood. In the instructions to the questionnaire, the subject was asked how often a particular symptom appeared in the 12 months following the traumatic event. It did not examine the extent to which they remained persistent later in life.

The Dissociative Experiences Scale—II [19]. The questionnaire comprises 28 statements describing aspects of different dissociative phenomena (acute dissociative symptoms, depersonalization, and derealization, as well as rare dissociative symptoms). Item example: “Some people have the experience of feeling that their body does not seem to belong to them.” The participant responds to the statements by marking on a 10-point scale how often they experienced the given dissociative symptom in the past year: from “never” (does not exhibit the given symptom) to “always” (exhibits given symptom). The current study used both the summary result scale and the detailed scales delineated according to symptom intensity: (1) depersonalization/derealization, (2) absorption, (3) distraction, (4) memory impairment (amnestic dissociation). Validation study for the original tool yielded satisfactory psychometric parameters (α = 0.95) [20].

Young Schema Questionnaire Short Form (YSQ-S3-PL) [21]; Young 1998; Oettingen, et al., 2018). The YSQ-S3-PL assesses the intensity of 18 early maladaptive schemas grouped into 5 domains. It consists of 90 items; the respondent marks their answer on a 6-point scale (1—completely untrue of me; 2—mostly untrue of me; 3—slightly more true than untrue; 4—moderately true of me; 5—mostly true of me; 6—describes me perfectly). Item example: “I do not feel capable of getting by on my own in everyday life”. The instrument has demonstrated satisfactory internal consistency and factor structure [21].

Procedure: The present study was questionnaire-based. Participation was voluntary and anonymous. Willingness to take part was compensated with a gift voucher. Participants were recruited at stationary and ambulatory facilities offering social and psychological support to victims of familial violence. Only individuals who were not in an acute phase of crisis related to their life circumstances were invited to the study. K-S test results showed that the distribution of most examined variables differed significantly from a normal distribution, which was taken into consideration when choosing appropriate statistical tests. All analyses were made using the IBM SPSS 27 statistical software.

## 3. Results

To identify the role of dissociative disorder symptoms in EMS aetiology, it was decided that statistical analysis was to be realized in three phases. During the first phase, using regression analysis, the effect of variables on the intensity of early maladaptive schemas was estimated. Then, in the second phase, correlation coefficients were calculated for the analysed variables. In the third phase, direct mediation analysis was done to verify the roles of the studied factors.

Regression analysis (Table 3) indicated that three factors play a role in the intensity of early maladaptive schemas: experienced childhood violence (β = 0.19; *p* < 0.01), symptoms of dissociative disorders (β = 0.17; *p* < 0.05), as well as being on pension for health reasons (β = 0.21; *p* < 0.05).

Correlation analysis results. The rho-Spearman test was chosen to analyse the correlation between all key variables, i.e., experiencing violence in childhood, dissociative symptoms (including subscales), and early maladaptive schemas (including their division into domains) (Table 4 and Table 5). Results of the correlation analysis reveal a link between the general intensity of violence and all subscales describing symptoms of dissociative disorders, including the overall severity of dissociative symptoms (rho = 0.17; *p* < 0.01), (rho = 0.17; *p* < 0.01), and more specifically depersonalization/derealization (rho = 0.15; *p* < 0.01), absorption (rho = 0.17; *p* < 0.01), distraction (rho = 0.14; *p* < 0.05), and amnestic dissociation (rho = 0.17; *p* < 0.01). Experiences of violence were also significantly correlated with the overall intensity of early maladaptive schemas (rho = 0.18; *p* < 0.01), as well as specifically with schemas from the domains of disconnection and rejection (rho = 0.30; *p* < 0.01), other-directedness (rho = 0.15; *p* < 0.01), and overvigilance and inhibition (rho = 0.15; *p* < 0.01). In turn, the overall severity of dissociative disorder symptoms correlated similarly with the overall intensity of early maladaptive schemas (rho = 0.22; *p* < 0,01), as well as specifically with schemas from the domains of disconnection and rejection (rho = 0.26; *p* < 0.01), impaired autonomy (rho = 0.18; *p* < 0.01), other-directedness (rho = 0.18; *p* < 0.01), and overvigilance and inhibition (rho = 0.22; *p* < 0.01).

**Figure 1:**
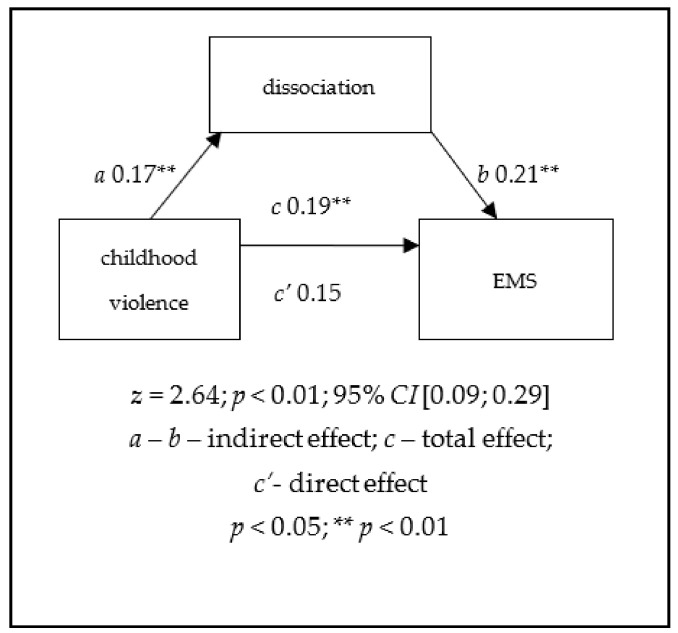
The mediating role of dissociation in the relationship between childhood violence and the intensity of early maladaptive schemas.

Mediation analysis results. In the second phase, direct mediation analysis was performed to verify the hypothesis regarding the dissociative disorders’ intermediatory role in the relationship between childhood violence and the intensity of early maladaptive schemas. Analyses were performed taking into account general EMS intensity (Figure 1), as well as levels of schema domains (Table 6). Analysis results confirmed the hypothesis that dissociative disorders play a mediating role in the development of early maladaptive schemas. Partial mediation occurred both for overall schema intensity (*a* = 0.17, *p* < 0.01, *b* = 0.21, *p* < 0.01, *c* = 0.19, *p* < 0.01, *c’* = 0.15, *p* < 0.01; *F* = 13.34, *p* < 0.01; *Z* = 2.64, *p* < 0.01; *CI* [0.09; 0.29]) and for the domains: disconnection and rejection (*a* = 0.17, *p* < 0.01, *b* = 0.22, *p* < 0.01, *c* = 0.30, *p* < 0.01, *c’* = 0.26, *p* < 0.01; *F* = 36.44, *p* < 0.01; *Z* = 2.69, *p* < 0.01; *CI* [0.20; 0.40]), other-directedness (*a* = 0.17, *p* < 0.01, *b* = 0.17, *p* < 0.01, *c* = 0.15, *p* < 0.01, *c’* = 0.12, *p* < 0.05; *F* = 8.44, *p* < 0.01; *Z* = 2.18, *p* < 0.05; *CI* [0.05; 0.25]), and overvigilance and inhibition (*a* = 0.17, *p* < 0.01, *b* = 0.22, *p* < 0.01, *c* = 0.15, *p* < 0.01, *c’* = 0.11, *p* < 0.05; *F* = 7.56, *p* < 0.01; *Z* = 2.19, *p* < 0.05; *CI* [0.04; 0.25]). Mediation was not found to be significant for domains: impaired autonomy (*a* = 0.17, *p* < 0.01, *b* = 0.18, *p* < 0.01, *c* = 0.03, *p* > 0.05, *c’*= 0.05, *p* > 0.05; *F* = 5.98, *p* < 0.01; *Z* = 2,19, *p* < 0.05; *CI* [−0.05; 0.16]) and impaired limits (*a* = 0.17 **, *p* < 0.01, *b* = 0.06, *p* > 0.05, *c* = 0.03, *p* > 0.05, *c’* = 0.02, *p* > 0.05; *F* = 0.36, *p* > 0.05; *Z* = 0.96, *p* > 0.05; *CI* [−0.07; 0.13]).

## 4. Discussion

The conducted research partially confirmed earlier ideas regarding the role of dissociative disorders in the psychological consequences of violence suffered in childhood. As assumed, experiencing violence in childhood leads to the development of early maladaptive schemas, especially those from the disconnection and rejection domain [11]. Mediation analysis indicates that this connection is strengthened when accounting for dissociative symptoms. This result suggests that a traumatic event can be recorded in the mind directly as a belief that other people are dangerous, i.e., will harm and exploit the individual. It can also be the case that the event is accompanied by dissociative symptoms and both factors influence the development of EMS, which become more severe than in the prior scenario. This makes the aforementioned symptoms indicators of serious psychopathological consequences. Similarly, dissociative symptoms can be observed in other patient populations with trauma experiences, e.g., among veterans or survivors of vehicle accidents [22]. These symptoms, especially in the form of depersonalization and derealization, may participate in the experienced traumatic event being rigidly recorded as early maladaptive schemas (i.e., a stable image of the self, the world, and other people), at the same time additionally impeding any modification to them including experiences that potentially altering the content of existing schemas could provide [13,14]. These circumstances could also seriously disrupt recognizing one’s own emotional states, as well as hinder discerning and assessing social situations—both those that are positive, which could influence and alter present schemas, and those that are negative, which could pose a real danger to the individual’s health and life [23]. For these reasons, the role of dissociative disorder symptoms is suspected to be crucial for the formation and solidifying of early maladaptive schemas.

The obtained results confirm the assumptions of the early maladaptive schema theory, which states that a given maladaptive schema develops under the influence of real-life experiences. Childhood experiences are particularly significant and set the direction for how future life experiences are interpreted. If beliefs comprising the schema develop sufficiently, they stabilize and solidify to create a tendency in the patient to interpret unclear situations in ways congruent with them or, if dissociative symptoms manifest, to make life choices that confirm their expectations [2]. In the analysed example, experiencing violence and neglect in childhood translated into the development of schemas from the disconnection and rejection domain.

Study limitations: The strength of the investigated relationship between experienced violence and the emergence of schemas from the disconnection and rejection domain suggests only a low level of dependence. The explanatory value of the mediation models was similarly low. This can be caused by one or both of the following factors: (1) retrospective measurements or (2) the nature of phenomena under study. The former is characteristic of all ex post facto studies, while the latter does require some explanation.

The mere occurrence of violence or serious neglect is not a sufficient condition of the development of early maladaptive schemas. It is assumed that other environmental factors are also significant, e.g., the level at which physical and emotional needs were met despite the experienced violence, the existence of stable relationships with others, or being provided support at crucial developmental moments. We must remember that certain individual properties may hold important roles as well, an example being temperamental traits [2,24]. Research on the interactions between temperament and experiences of severe stress indicate that certain temperamental traits (e.g., briskness and endurance) can predispose the individual to better cope emotionally with traumatic events. On the other hand, high perseveration and emotional reactivity can exacerbate their negative psychological consequences [25]. Accounting for and controlling a larger number of both environmental and individual factors could make it possible to identify the significance of each variable for the emergence of EMS, thus also verifying the strength of dependencies between the studied variables. Conclusion: The results of the study show that childhood violence experiences lead to an increase in early maladaptive schemas. The mediation analysis confirmed that the occurrence of dissociative disorder symptoms in the aftermath of experienced violence can further intensify the formation of EMS. The results confirm previous assumptions about the role that dissociative symptoms play in changes in personality as a consequence of experiencing early traumatic events.

## Figures and Tables

**Table 1:** Characteristics of the study participants, differences in childhood abuse (*N* = 342).

Variable	Abuse Experience Period; Mean Rank		Mann–Whitney *U*	*Z*	*p*	Cohen’s *d*
	Childhood (*N* = 141)	Childhood and Adulthood (*N* = 201)				
childhood violence- overall intensity	169.04	173.23	13,823.50	−0.39	0.70	-
emotional violence	183.48	163.09	12,481.00	−1.88	0.06	-
physical violence	169.67	172.79	13,912.00	−0.29	0.77	-
sexual violence	165.89	175.44	13,379.00	−0.93	0.35	-
emotional neglect	165.96	175.39	13,389.50	−0.87	0.38	-
physical neglect	157.96	181.00	12,261.50	−2.13	0.03	0.23

**Table 2:** Characteristics of the study participants’ education, relationship, and employment status (*N* = 342).

Variable		
Age	*M*	Range
metrical	35.8	18–65
when violence occurred in childhood	7.1	4–12
when violence occurred in adulthood	24.64	18–45
Education	%	*N*
higher	20.2	69
high-school	29.8	102
vocational	19.6	67
elementary	20.8	71
no data	9.6	33
Relationship status	%	*N*
never married	23.2	89
married	20.3	78
cohabitation	21.9	84
separated	6.5	25
divorced	13.8	53
widow	1.8	7
other	0.6	2
no data	12	46
Employment status	*M*	*N*
paid work	39.2	134
homemaker	11.4	39
student	6.7	23
retired	1.2	4
pension	4.1	14
unemployment	25.1	86
other	5.9	20
no data	6.4	22

**Table 3:** The impact of the studied variables on the early maladaptive schemas. Regression analysis results (*N* = 342).

Variable	B	Std. Error	*β*	*p*
schemas—overall intensity (YSQ)				
Violence—overall intensity (CTQ)	0.17	0.06	0.19	<0.01 **
dissociative disorders—overall intensity (DES)	0.16	0.06	0.17	0.02 *
age	0.00	0.08	0.00	0.98
age of childhood violence	−0.01	0.02	−0.20	0.84
Relationship status				
single	0.15	0.37	0.07	0.68
married	0.07	0.37	0.03	0.85
cohabitation	0.04	0.38	0.01	0.92
separation	0.08	0.41	0.02	0.84
divorce	0.18	0.38	0.07	0.63
widow	−0.51	0.63	−0.07	0.42
Employment				
full-time job	0.17	0.35	0.09	0.64
housewife	0.38	0.37	0.12	0.30
student	−0.08	0.44	−0.01	0.85
retired	0.63	0.61	0.10	0.30
pensioner for health reasons	1.12	0.50	0.21	0.03 *
unemployed	0.18	0.33	0.08	0.59
Education				
Academic degree	0.07	0.70	0.03	0.92
high-school	−0.31	0.70	−0.15	0.66
vocational	−0.14	0.69	−0.06	0.84
elementary	−0.18	0.70	−0.08	0.80
*R^2^* = 0.14. *F* = 19.69 *				

* *p* < 0.05; ** *p* < 0.01.

**Table 4:** Results of analysing the relationships between violence in childhood. Early maladaptive schemas and symptoms of dissociation (*N* = 342). rho-Spearman correlation coefficient.

Variable	Dissociative Disorders—Overall Intensity (DES)	Depersonalization/Derealization	Absorption	Distraction	Memory Impairment
violence—overall intensity (CTQ)	0.17 **	0.15 *	0.17 **	0.14 *	0.17 **
schemas—overall intensity (YSQ)	0.22 **	0.20 **	0.22 **	0.24 **	0.17 **

* *p* < 0.05; ** *p* < 0.01.

**Table 5:** Results of analysing the relationships between childhood violence. Early maladaptive schemas (domains) and dissociation symptoms (*N* = 342). rho-Spearman correlation coefficient.

Variable	Violence—Overall Intensity (CTQ)
Early maladaptive schemas—domains (YSQ)	
disconnection and rejection domain	0.30 **
impaired autonomy domain	0.07
impaired limits domain	−0.01
other-directedness domain	0.15 **
overvigilance and inhibition domain	0.15 **
Dissociative disorders symptoms (DES)	
depersonalization/ derealization	0.15 *
absorption	0.17 **
distraction	0.14 *
memory impairment	0.17 **

* *p* < 0.05; ** *p* < 0.01.

**Table 6:** Mediating role of dissociation in the relationship between childhood violence and the intensity of early maladaptive schemas.

	Paths				Model Summary			Sobel Test		95% *CI*	
	*a*	*b*	*c*	*c*′	*R^2^*	*F*	*p*	*Z*	*p*	Lower	Upper
**Schema domains**											
overall intensity	0.17 **	0.21 **	0.19 **	0.15 **	0.04	13.34	<0.01	2.64	<0.01	0.09	0.29
disconnection and rejection domain	0.17 **	0.22 **	0.30 **	0.26 **	0.11	36.44	<0.01	2.69	<0.01	0.20	0.40
impaired autonomy domain	0.17 **	0.18 **	0.03	0.05	0.04	5.98	<0.01	2.19	<0.05	−0.05	0.16
impaired limits domain	0.17 **	0.06	0.03	0.02	0.00	0.36	>0.05	0.96	>0.05	−0.07	0.13
other-directedness domain	0.17 **	0.17 **	0.15 **	0.12 *	0.03	8.44	<0.01	2.18	<0.05	0.05	0.25
overvigilance and inhibition domain	0.17 **	0.22 **	0.15 **	0.11 *	0.02	7.56	<0.01	2.19	<0.05	0.04	0.25

* *p* < 0.05; ** *p* < 0.01.

## Data Availability

The data presented in this study are available on request from the corresponding author. The data are not publicly available due to legal and privacy issues.

## References

[1] Arntz A., van Genderen H. (2016). Terapia Schematów w Zaburzeniu Osobowości Typu Borderline.

[2] Young J.E., Klosko J.S., Weishaar M.E. (2014). Terapia Schematów. Przewodnik Praktyka.

[3] Carmen E., Rieker P.P., Mills T., Rieker P.P., Carmen E. (1984). Victims of Violence and Psychiatric Illness. The Gender Gap in Psychotherapy.

[4] Izdebska A., Beisert M. (2021). The level of personality organization and revictimization in lives of child sexual abuse survivors. J. Interpers. Violence.

[5] Mauricio A.M., Tein J.Y., Lopez F.G. (2007). Borderline and antisocial personality scores as mediators between attachment and intimate partner violence. Violence Vict..

[6] Ørke E.C., Vatnar S.K.B., Bjørkly S. (2018). Risk for Revictimization of Intimate Partner Violence by Multiple Partners: A Systematic Review. J. Fam. Violence.

[7] Zanarini M.C., Frankenburg F.R., Reich D.B., Marino M.F., Haynes M.C., Gunderson J.G. (1999). Violence in the Lives of Adult Borderline Patients. J. Nerv. Ment. Dis..

[8] Aaron D.J. (2013). Early maladaptive schemas and substance use: Implications for assessment and treatment. Addict. Disord. Treat..

[9] Carr S.N., Francis A.J.P. (2010). Early maladaptive schemas and personality disorder symptoms: An examination in a non-clinical sample. Psychol. Psychother. Theory, Res. Pract..

[10] Corral C., Calvete E. (2014). Early Maladaptive Schemas and Personality Disorder Traits in Perpetrators of Intimate Partner Violence. Span. J. Psychol..

[11] Gay L.E., Harding H.G., Jackson J.L., Burns E.E., Baker B.D. (2013). Attachment Style and Early Maladaptive Schemas as Mediators of the Relationship between Childhood Emotional Abuse and Intimate Partner Violence. J. Aggress. Maltreat. Trauma.

[12] Rezaei M., Ghazanfari F. (2016). The role of childhood trauma, early maladaptive schemas, emotional schemas and experimental avoidance on depression: A structural equation modeling. Psychiatry Res..

[13] Chu J.A. (1992). The revictimization of adult women with histories of childhood abuse. J. Psychother. Pract. Res..

[14] Kluft R.P., Michelson L.K., Ray W.J. (1996). Dissociative Identity Disorder. Handbook of Dissociation: Theoretical, Empirical, and Clinical Perspectives.

[15] Risser H.J., Hetzel-Riggin M.D., Thomsen C.J., McCanne T.R. (2006). PTSD as a mediator of sexual revictimization: The role of reexperiencing, avoidance, and arousal symptoms. J. Trauma. Stress.

[16] Acierno R., Resnick H., Kilpatrick D., Saunders B., Best C. (1999). Risk Factors for Rape, Physical Assault, and Posttraumatic Stress Disorder in Women: Examination of Differential Multivariate Relationships. J. Anxiety Disord..

[17] Tjaden P.G., Thoennes N. (2000). Extent, Nature, and Consequences of Intimate Partner Violence: Findings from the National Violence against Women Survey.

[18] Bernstein D.P., Fink L., Handelsman L., Foote J. (1998). Childhood Trauma Questionnaire. Assessment of Family Violence: A Handbook for Researchers and Practitioners.

[19] Carlson E.B., Putnam F.W. (1993). An update on the Dissociative Experience Scale. Dissociation.

[20] Frischholz E.J., Braun B.G., Sachs R.G., Hopkins L., Shaeffer D.M., Lewis J., Leavitt F., Pasquotto J.N., Schwartz D.R. (1990). The Dissociative Experiences Scale: Further replication and validation. Dissociation Prog. Dissociative Disord..

[21] Oettingen J., Chodkiewicz J., Mącik D., Gruszczyńska E. (2018). Polska adaptacja i walidacja krótkiej wersji Kwestionariusza Schematów Younga (YSQ-S3-PL). Psychiatr. Pol..

[22] van der Kolk B.A. (2003). Psychological Trauma.

[23] Gabbard G.O., Michels R. (2014). Psychodynamic Psychiatry in Clinical Practice.

[24] Berzenski S.R., Yates T.M. (2010). A Developmental Process Analysis of the Contribution of Childhood Emotional Abuse to Relationship Violence. J. Aggress. Maltreat. Trauma.

[25] Strelau J., Zawadzki B. (2005). Trauma and temperament as predictors of intensity of posttraumatic stress disorder symptoms after disaster. Eur. Psychol..

